# KIs Virus and Blood Donors, France

**DOI:** 10.3201/eid1808.120442

**Published:** 2012-08

**Authors:** Philippe Biagini, Mhammed Touinssi, Vital Galicher, Philippe de Micco

**Affiliations:** Etablissement Français du Sang Alpes-Méditerranée et Aix-Marseille Université, Marseille, France

**Keywords:** KIs virus, KIs-V, blood donors, viruses, France, hepatitis E virus, Bunyaviridae, anellovirus

**To the Editor:** KIs virus (KIs-V) is a putative new virus identified recently in the blood of persons in Japan (*1*). The first partial sequence of KIs-V was characterized unexpectedly, when PCR primers were used that were directed primarily to the consensus domain of helicase of positive-stranded RNA viruses. Extensive physicochemical and molecular analysis suggested that KIs-V is an enveloped virus with a circular, double-stranded DNA genome of ≈9,500 bp (prototype isolate: GenBank accession no. AB550431); its genetic diversity is presumed to be extremely low because the 4 complete genomes already characterized in Japan harbor strict identical sequences. The 13 potential genes identified by in silico analysis exhibit an overall low sequence homology to other known viral proteins (*1*). Until now, KIs-V epidemiologic data have been related only to the original study in which the authors analyzed plasma samples from 516 blood donors categorized into 4 groups by alanine aminotransferase (ALT) level (either <60 IU/L or >60 IU/L) and the presence or absence of hepatitis E virus (HEV) antibodies. As a result, KIs-V DNA was detectable at elevated prevalence in the high ALT level/HEV antibody–positive group (36%, n = 100); viral loads, checked for a few samples, ranged from 10^6^ to 10^8^ copies/mL. KIs-V DNA also was identified in HEV antibody–negative samples, with low or high ALT level (≈0.8%, n = 120, and 1%, n = 100, respectively) (*1*).

To gain insights about the potential presence of this virus in the blood of persons in France, we investigated KIs-V DNA in the plasma of 576 healthy blood donors (mean age 40 years; 306 men; men:women 1:1.13). Blood samples were collected in vacuum tubes (Vacutainer, SST, Becton Dickinson, Meylan, France) and centrifuged, and plasma aliquots were stored at −80°C until use. Nucleic acids were extracted from 1-mL plasma volumes (MagNA Pure LC, Roche Diagnostics, Meylan, France) (*2*) and tested for KIs-V DNA by using the same nested PCR system for screening Japanese blood donors (*1*). Briefly, one tenth (5 μL) of extracted nucleic acids were first amplified by using primers 101-C (5′-CAACACCGCAATCACAAAGT-3′) and N101-B (5′-AACATTGAAACGTCATGTCC-3′) (0.8 μM each) in a 50-μL mix containing deoxynucleotide triphosphates (0.2 mM each) (Roche) and 2 U Taq DNA polymerase (Invitrogen, Cergy Pontoise, France) with its corresponding buffer. One microliter of the amplification mixture was subsequently used in a second-round PCR with primers KS-2 (5′-CTCGTCCTCGTCGTCATCGTA-3′) and N101-D (5′-CATTTGCTCCCGCTGGAGATG-3′) under the same conditions as above. The amplification conditions for first- and second-round PCR were 94°C for 3 min, followed by 40 cycles of 94°C for 30 s, 55°C for 30 s, and 72°C for 2 min. Expected amplification products were 458 bp (PCR-1) and 304 bp (PCR-2). Using dilutions of a synthetic template corresponding to the target sequence, we estimated the sensitivity of the amplification assay to be <5 copies of target sequence by limiting-dilution assay.

Negative (sterile water) and positive controls (synthetic template dilutions) were added systematically to each amplification run. A PCR control intended to check the quality of the nucleic acids extraction procedure was also performed systematically on 4 randomly selected samples of each batch (n = 32); this control was based on the detection of an extremely prevalent DNA virus (Torque Teno virus and related viruses, family *Anelloviridae*) by using a highly conserved amplification system (*3*).

Among the 576 plasma samples tested, no positive signal was identified for KIs-V DNA after agarose gel electrophoresis of PCR-1 and PCR-2. Amplification controls (negative, positive, anellovirus*e* DNA) confirmed the validity of these results.

Using the PCR detection system adopted by Satoh et al., combined with the extraction of large plasma volumes, we were not able to detect KIs-V DNA in the blood of donors tested, suggesting an uncommon frequency in healthy persons in France. Information related to HEV status or ALT levels were not available here because both parameters are not evaluated for routine blood donor screening in France; HEV seroprevalence studies involving blood donors from northern and southwestern France indicate discrepant results (≈3%–≈52%, IgG), possibly related to serologic assay performances and/or geographic differences (*4*). The precise identity of KIs-V remains uncertain, but according to its extensive initial characterization, complementary studies probably will confirm its viral origin. Molecular characterization of new full-length sequences will be needed to investigate the real genetic diversity of KIs-V and to help design optimized molecular detection systems.

The implication of KIs-V in human health remains under debate. The original publication highlighted the fact that HEV antibody–positive persons in Japan who had moderately elevated ALT levels at a prevalence of KIs-V infection that is non-negligible; such findings could suggest a link between the virus and liver dysfunctions. HEV and KIs-V also could share the same route of contamination, i.e., foods (*5*). Further investigations involving diverse human cohorts need to be undertaken to better understand the natural history of KIs-V in humans.
